# Retention and Internal Loading of Phosphorus in Shallow, Eutrophic Lakes

**DOI:** 10.1100/tsw.2001.72

**Published:** 2001-08-23

**Authors:** Martin Sondergaard, Peder Jens Jensen, Erik Jeppesen

**Affiliations:** National Environmental Research Institute, Department of Lake and Estuarine Ecology, Vejlsøvej 25, P.O. Box 314, DK-8600 Silkeborg, Denmark

**Keywords:** lake recovery, sediment, phosphorus release, phosphorus fractionation, lake restoration

## Abstract

This paper gives a general overview of the nature and important mechanisms behind internal loading of phosphorus (P), which is a phenomenon appearing frequently in shallow, eutrophic lakes upon a reduction of the external loading. Lake water quality is therefore not improved as expected. In particular summer concentrations rise and P retention may be negative during most of the summer. The P release originates from a pool accumulated in the sediment when the external loading was high. In most lake sediments, P bound to redox-sensitive iron compounds or P fixed in more or less labile organic forms constitute major fractions forms that are potentially mobile and eventually may be released to the lake water. The duration of the recovery period following P loading reduction depends on the loading history, but it may last for decades in lakes with a high sediment P accumulation. During the phase of recovery, both the duration and net P release rates from the sediment seem to decline progressively. Internal P loading is highly influenced by the biological structure as illustrated by lakes shifting from the turbid to the clearwater state as a result of, for example, biomanipulation. In these lakes P concentrations may be reduced to 50% of the pre-biomanipulation level and the period with negative retention during summer can thus be reduced considerably. The duration of internal loading can be reduced significantly by different restoration methods such as dredging to remove accumulated P or addition of iron or alum to elevate the sorption capacity of sediments. However, an important prerequisite for achieving long-term benefits to water quality is a sufficient reduction of the external P loading.

## TABLE OF CONTENTS

INTRODUCTION
RETENTION OF PHOSPHORUS
FORMS OF PHOSPHORUS IN THE SEDIMENT
RELEASE MECHANISMS
SEASONALITY OF INTERNAL LOADING AND RETENTION
LONGEVITY OF INTERNAL LOADING
IMPORTANCE OF BIOLOGICAL STRUCTURE
METHODS TO OVERCOME INTERNAL LOADING
ACKNOWLEDGMENT
REFERENCES

## INTRODUCTION

Nutrients, and in particular phosphorus (P) availability, have long been recognized as a factor of paramount importance for the water quality of lakes[1,2,3,4,5]. If P loading is excessive, phytoplankton is favored and this has significant negative implications for the overall water quality and biodiversity of the lake: the water becomes turbid, toxic algae may develop, submerged macrophytes disappear, fish stocks change toward less desirable species, and top-down control by zooplankton on phytoplankton decreases.

To reverse the eutrophication process, multiple measures have been introduced worldwide during the last 50 years with the aim to reduce the external loading of lakes, both at point sources by establishing sewage works and by reducing the nutrient loading from arable soils[6,7,8]. Improved nutrient removal and catchment retention have also been achieved through the establishment of new wetlands or re-meandering of channelized streams[7,9,10].

Although some lakes respond rapidly to changes in external P loading[11], lake recovery following a reduction of external loading is often delayed[12,13,14]. Accumulated P in the sediment during the period of high loading equilibrates with the new and reduced loading and is released into the lake water[11,12,15,16]. This internal loading can be so significant that it prevents improvements in water quality and the lake may thus not meet the established water quality criteria[17,18] *despite* a reduction of the P-loading to a level where improvements were expected.

Originally, mainly stratified lakes, which develop an anoxic hypolimnion during summer, were believed to suffer from internal P loading due to the redox-dependent release of iron-bound P[19,20,21,22,23]. In contrast, well-oxidized conditions throughout the water column and throughout the season in shallow lakes were believed to always establish an oxidized layer in the surface sediment that prevented P release. Later, numerous studies showed that sediments also release P if the overlaying water is aerobic[22,24,25,26] and that P released from the sediment of shallow lakes can constitute a substantial part of the total loading and sometimes even exceeds the external loading of P[16,27,28]. As a further difference to deep lakes, the sediment of shallow lakes may be in direct contact with the photic zone during the whole season that, together with a higher sediment surface per volume of water as water depth decreases, increases the importance of sediment–water interactions particularly in shallow lakes. The regular mixing regime in shallow lakes guarantees stable and near optimum conditions for primary production[29].

In this review we discuss the retention and release mechanisms of P in shallow, temperate lakes and illustrate it with results obtained from our own work in Danish, mainly shallow and eutrophic, lakes. In many of the lakes the external loading of P recently has been reduced but internal loading from the sediment constitutes a severe problem when trying to improve lake water quality.

## RETENTION OF PHOSPHORUS

P enters the lake in either a particulate form, which can be directly deposited in the sediment, or as dissolved phosphate, which can be incorporated in organic matter by primary producers that eventually sink to the bottom in an organic form (Fig. 1). Sedimentation may also occur via co-precipitation with calcium carbonate[30,31,32,33] or the formation of and adsorption by iron hydroxides[32,34,35,36,37]. Having reached the sediment, P becomes a constituent part of numerous chemically and biologically mediated processes and is ultimately permanently deposited in the sediment or released and returned to the water column via the interstitial water. Between these two terminal stages, P may be transformed several times and incorporated into different organic and inorganic P compounds (see below). During early diagenesis, most of the sedimenting particulate P may be redissolved[38].

The retention of P is lake specific. Lakes with a high flushing rate tend to have a lower relative P retention than lakes with a slower flushing rate. This relationship has been demonstrated through different simple, empirically established steady-state models of the Vollenweider type: $P_{lake} = L_P/(z^*(\sigma + \rho_w)$, relating in-lake P ($P_{lake}$) to specific loading ($L_P$), sedimentary loss ($\sigma$), lake mean depth (z), and flushing rate ($\rho_w$)[39,40]. Sedimentation rate can be estimated on the basis of observed empirical relationships with hydraulic retention time. As these models are based on systems in equilibrium, however, they cannot adequately describe the transient phase following a loading reduction before a new equilibrium is established. This is illustrated by the discrepancy often seen between measured and calculated annual P retention in eutrophic lakes in recovery[16,41]. Attempts have been made to extend the Vollenweider models with simple models for P retention in lake sediment[28], or more or less complex dynamic models that attempt to describe the kinetics of the numerous physical, chemical, and biological processes determining sediment release[42,43,44,45]. Quantification of the processes behind the equilibration and recovery period is, however, generally not well described, which impedes the prediction of future lake water quality in these lakes.

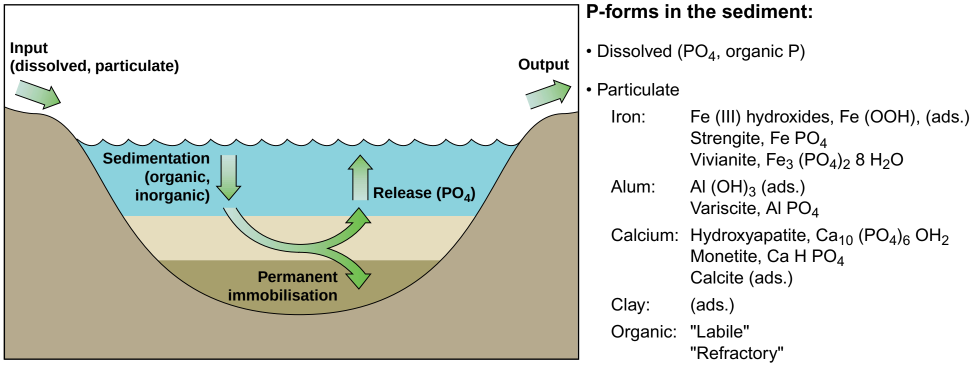

**FIGURE 1.** Conceptual diagram showing input/output of phosphorus and some of the most important pathways and phosphorus compounds in the sediment and water in shallow lakes. From the input to the output or the immobilization and permanent deposition in the sediment, phosphorus is transformed between different organic and inorganic fractions. ads. = adsorption.

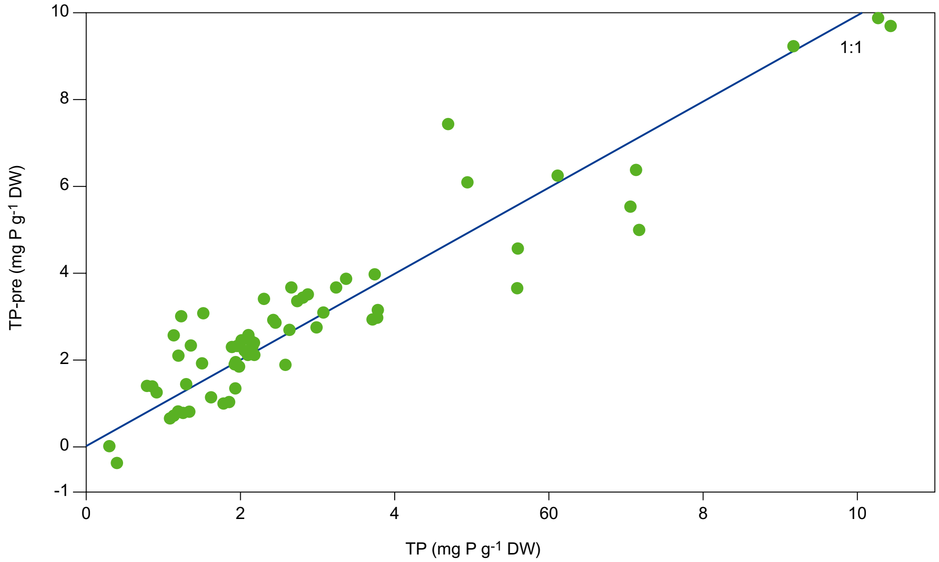

**FIGURE 2.** Measured total phosphorus in the surface sediment ($TP_{sed}$) vs. calculated total phosphorus in the surface sediment ($TP_{sed}$) of 21 Danish lakes (each lake represented by 2 to 5 different locations). The equation can be described as $TP_{sed}$ = -2,46 + 0.26*Pex + 0.031*Fe + 0.0090*LOI + 0.58*z, $r^2 = 0.91$, $p < 0.0001$, where Pex is the external phosphorus load (g P $m^{-2}$ $y^{-1}$), Fe is the concentration of total iron (mg Fe $g^{-1}$ DW), LOI is loss on ignition and z the mean water depth. Fe alone explain 36% and Fe and Pex 68% of the variability. Reprinted from *Water Research*, Vol. 30, Søndergaard, M., Windolf, J., and Jeppesen, E., Phosphorus fractions and profiles in the sediment of shallow Danish lakes as related to phosphorus load, sediment composition and lake chemistry. ©1996, with permission from Elsevier Science.

The combined ferric oxides and hydroxides available in the sediment may bind phosphate very effectively. The involvement of iron in the dynamic equilibrium between the sediment and water has led to the suggestion that an iron-dependent threshold exists for the sediment's ability to sorp P. Experiments from shallow lakes have shown the retention capacity to be high as long as the Fe/P ratio exceeds 15 (by weight), and when above this ratio internal P loading may be prevented by keeping the surface sediment oxidized[46]. The likely presence of a threshold is supported by the strong positive relationship existing between measured concentrations of P in the surface sediment and predicted concentrations based on iron concentrations and P loading (Fig. 2).

## FORMS OF PHOSPHORUS IN THE SEDIMENT

Fixation of P in the sediment depends on the transport of soluble phosphate between solid components, adsorption-desorption mechanisms, chemosorption, and biological assimilation[47]. Chemosorption is the chemical fixation of soluble compounds that are subsequently unaffected by changes in solute concentrations, whereas adsorption is a physical fixation of soluble compounds on surfaces in constant equilibrium with solute concentrations. Ad- and chemosorption processes often depend on both pH and the redox potential and are therefore influenced by the bacterial metabolism.

Fractionation schemes using different methods of chemical sequential extractions have been widely used in order to describe the many different forms in which P can be found in the sediment[48,49,50,51]. Typically, these characterize P as being bound to a variety of organic and inorganic sediment components (Fig. 1). The inorganic forms are often bound to iron, aluminum and calcium compounds or to clay minerals[22,52]. The organic P occurs in more or less labile forms or in a refractory form that is not released during mineralization and constitutes a fraction permanently buried in the sediment. It can be debated which type of sediment P the different fractionations actually measure[53,54], but fractionation schemes usually yield operationally defined fractions[53].

The reason for fractionating and studying P forms in the sediment is usually to allow a more precise description of the potentials for P release from the sediment and to predict its future influence on lake water concentrations[55,56]. Particularly loosely sorbed organic and inorganic fractions as well as the iron-bound and redox-sensitive sorption of P are considered potentially mobile, which may contribute to an internal release[22,57,58,59,60,61]. Total P release rates have been found to be closely correlated to the iron-bound P components in the sediment[62]. Due to inadequate knowledge of the mechanisms behind internal loading in shallow lakes[63,64], however, it has been difficult thus far to establish general relationships between simple lake or sediment characteristics, including different sediment P forms and the intensity and duration of internal loading. Such knowledge may provide information on the overall and long-term conditions for P sorption expected to prevail in the sediment, whereas knowledge of static P binding gives only limited insight into the changes of P forms released under dynamic conditions.

## RELEASE MECHANISMS

The exchange of P between water and sediment is influenced by many factors. These include biological (e.g., bacterial activity, mineralization processes, and bioturbation), chemical (e.g., redox conditions, pH, iron:P ratio, nitrate availability), and physical factors (e.g., resuspension and sediment mixing)[22,25,28,64,65,66,67,68]. Overall, the net release of P observed from a sediment is the difference between the downward flux caused mainly by sedimentation of particles continuously produced in the water column (algae, detritus) and the upward flux and gross release of P driven by the decomposition of organic matter and the P gradients and transport mechanisms established in the sediment. Laboratory experiments often demonstrate high release rates compared to *in situ* or mass balance calculations because the downward flux via sedimentation is excluded. The significance of gross release vs. net release can be observed in lakes during periods with clear water and low sedimentation rates. Here, in the absence of sedimentation, a continuously high release rate from the sediment can establish high P concentrations in the lake water within a few days (Fig. 3).

The interstitial water of the sediment that normally contains less than 1% of the sediment's total P pool is important for the P transport between sediment and water. Interstitial phosphate constitutes the direct link to the water phase above and the solid-liquid phase boundary between water and sediment[22,69]. An upward transport of P is created via a diffusion-mediated concentration gradient, normally appearing just below the sediment surface. Bioturbation from benthic invertebrates or through gas bubbles produced in deeper sediment layers during the microbial decomposition of organic matter may significantly enhance the process[63,70,71,72]. Benthic invertebrates can also have a negative effect on P release by supplying oxic water into the sediment and increasing the depth of the oxidized layer[22].

Wind-induced resuspension may significantly affect the sediment P release, particularly in shallow lakes. In very shallow lakes, resuspension events increase, more or less continuously, the contact between sediment and water[73,74], and particulate nutrients settling to the bottom are most probably resuspended several times before long-term burial[75]. In some lakes, year-to-year variation in internal P loading has been shown to be largely controlled by wind mixing[76]. Potentially, resuspension of sediment can both reduce and increase the sediment P release because the overall process depends on the actual equilibrium conditions between sediment and water[67,75,77].

Submerged macrophytes, which particularly in shallow lakes may become abundant and the plant-filled volume consequently high, may also influence the P cycle both negatively and positively. Oxygen released from the roots can increase the redox-sensitive P sorption to iron compounds[78,79]

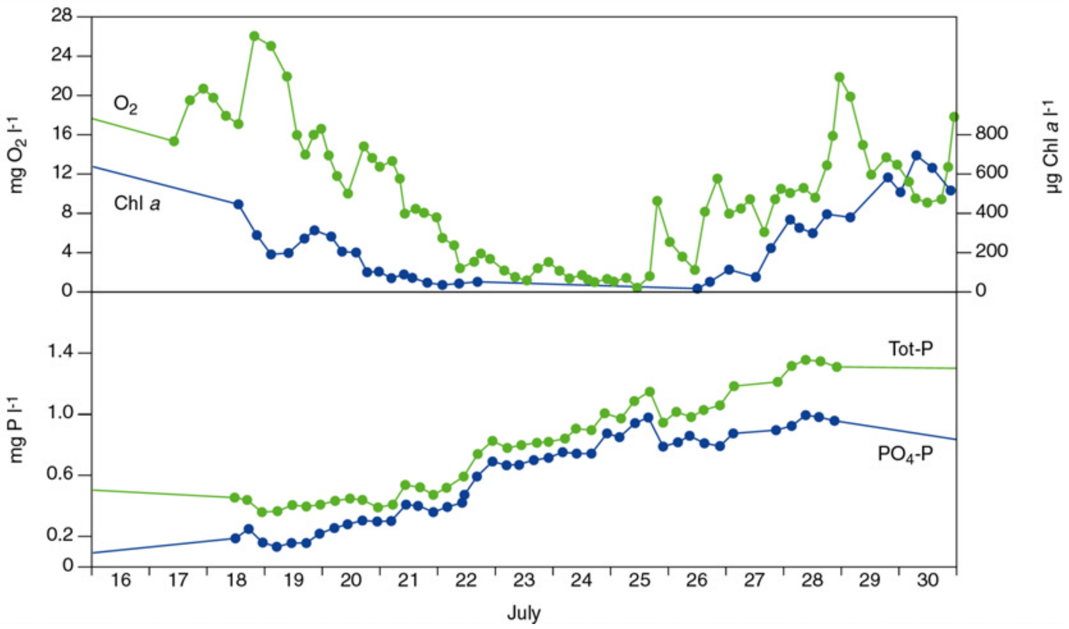

**FIGURE 3.** Changes in total phosphorus and dissolved phosphate in hypertrophic Lake Søbygaard in July 1985. The phytoplankton biomass decreased from above 800 to less than 50 µg chl *a* within 5 days reaching a minimum of 12 µg chl *a* $l^{-1}$. During the event, which lasted for 3–5 days, total phosphorus and dissolved phosphate in the lake increased correspondingly from 0.24 to 0.96 mg P $l^{-1}$ and from 0.37 to 1.33 mg P $l^{-1}$. Corrected for input and output to the lake, the average net release rate for an 8-day period was 145 mg P $m^{-2}$ $d^{-1}$. From Søndergaard, M., Jeppesen, E., Kristensen, P., and Sortkjær, O. (1990) Interactions between sediment and water in a shallow and hypertrophic lake: a study on phytoplankton collapses in Lake Søbygaard, Denmark. *Hydrobiologia* **191**, 139-148. With kind permission from Kluwer Academic Publishers. URL: http://www.wkap.nl and high abundance of macrophytes may diminish the resuspension rate and decrease P release from the sediment[80,81]. Increased P release may be recorded in dense macrophyte beds and beneath macrophyte canopies due to low oxygen concentrations[82,83].

## SEASONALITY OF INTERNAL LOADING AND RETENTION

P concentrations often increase during summer in shallow, temperate lakes[11,15,63,64,75,84,85]. One explanation is increased inlet concentrations because waste water constitutes a larger proportion during summer at low-river discharge[73]. However, in most cases the increase can only be the result of increased sediment loading, and summer P concentrations thus seem to be largely controlled by internal processes[16,26,86,87]. The most pronounced impact is often found in the most eutrophic lakes (Fig. 4).

P retention exhibits a seasonal pattern that mimics the seasonal variation in lake P (Fig. 5). During winter retention is positive while it is negative during part of the summer. Even less eutrophic lakes exhibit a period with negative retention in mid-summer, but the duration of negative retention increases with increasing total P concentrations. A highly negative retention occurring in May in the most eutrophic lakes suggests that the onset of the increasing biological activity in spring triggers the release of some of the P retained during winter.

Several explanations may be offered for the seasonal variation in the sediment's capacity to retain P and its dependency on the eutrophication level, but the strong seasonality indicates that the background mechanisms are linked to temperature and biological activity[16,25,28]. These include stimulation of organic matter mineralization and the release of inorganic phosphate as temperature increases[22,26], photosynthetically elevated pH in eutrophic lakes increasing release rates from

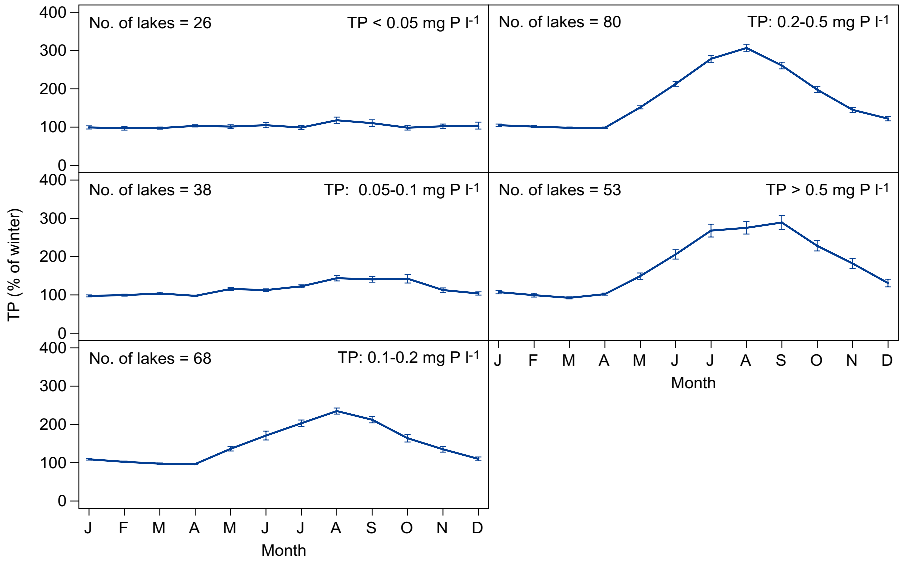

**FIGURE 4.** Seasonal variation in total phosphorus concentrations as percentage of winter values (1 Jan to 31 March) in 265 Danish lakes with different categories of mean summer total phosphorus (TP). Seasonal phosphorus variations were highly related to nutrient levels. In lakes with a TP below 0.05 mg P $l^{-1}$, seasonal variation was low and summer concentrations did not differ much from winter values, whereas in more eutrophic systems, and particularly when TP was above 0.1 mg P $l^{-1}$, summer concentrations were significantly and typically two- to fourfold higher than winter values. The lakes included in this analysis were mainly eutrophic (half of the lakes with a mean summer total phosphorus between 0.15 and 0.58 mg P $l^{-1}$), shallow (half of the lakes with a mean depth between 1.2 and 3.2 m) and relatively small (half of the lakes with an area between 17 and 137 ha). The lakes were sampled at least 10 times annually for the past 10-15 years. Each lake is only represented once. Only epilimnic (surface) samples were included. Modified from Jeppesen et al.[26], and Søndergaard et al.[16].

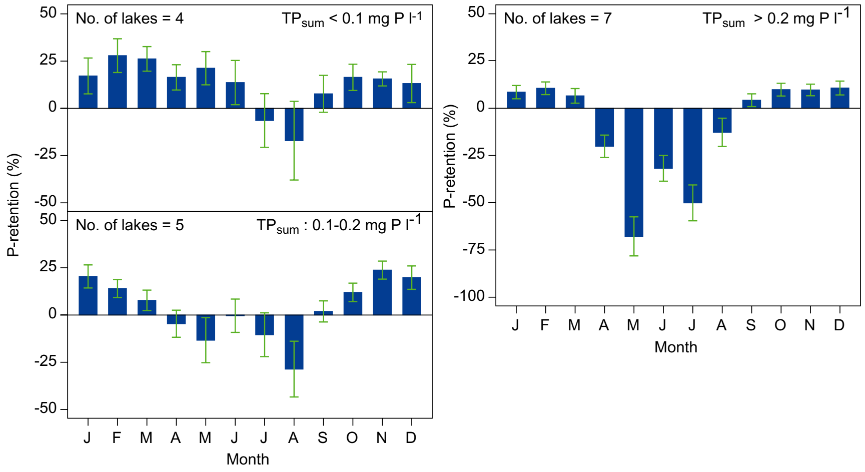

**FIGURE 5.** Seasonal phosphorus retention within three different categories of mean summer concentrations of total phosphorus (TP) in 16 Danish lakes measured for 8 years. Seasonally, large differences were recorded between eutrophic and less eutrophic lakes. In lakes with a TP below 0.1 mg P $l^{-1}$, mean phosphorus retention was positive throughout the year excepting July and August, while retention was negative from April to September in lakes with a TP > 0.1 mg P $l^{-1}$. Retention was most negative in May and July (as high as 50–65% of external loading), while in June retention was often less negative, particularly in lakes with a TP between 0.1 and 0.2 mg P $l^{-1}$. All the lakes were relatively small, turbid, eutrophic and with a short hydraulic retention time. To establish monthly mass balances the main inlet of each lake was sampled 18–26 times annually, depending on seasonal variations in discharge, while the minor inlets were sampled less frequently, depending on their relative contribution to total loading. Outlet samples were collected twice monthly during summer and once monthly during winter, i.e., 19 times annually. The lakes represent various loading histories, but in most of the lakes the loading has been reduced within the past 10–15 years. From Søndergaard, M., Jensen, J.P., and Jeppesen, E. (1999) Internal phosphorus loading in shallow Danish lakes. *Hydrobiologia* **408/409**, 145-152. With kind permission from Kluwer Academic Publishers. URL: http://www.wkap.nl iron-bound P[64,88,89,90], and increased sedimentation of organic material related to the seasonal variation in phytoplankton productivity[15,90] which, at higher temperatures, reduces the capacity of the uppermost sediment to retain P. In addition, as organic loading increases and mineralization processes are strengthened, the penetration depth into the sediment of oxygen and nitrate declines[25,91]. Jensen and Andersen[25] observed that the temperature effect on P release was highest in lakes with a large proportion of iron-bound P and they noticed a decrease in the thickness of the oxidized surface layer with increasing temperatures, suggesting a redox-sensitive release. They, however, also showed that whereas the presence of nitrate during winter and early summer diminished the release rates, nitrate addition in late summer enhanced P release in the same lakes, probably by stimulating the mineralization process. If nitrate is consumed and high sulphate concentrations and a supply of biodegradable organic matter persist, desulphurication and sulphur cycling may become an important part of the sediment processes[92]. Hydrogen sulphide formed from sulphate reduction causes the reduction of Fe(III) and the formation of iron sulphide and decreases the potential of P sorption, factors that may increase the release of P from the sediment[37,61,63,93]. In this situation the internal dynamic P release from lake sediments is determined by the ligand exchange of phosphate against sulphide with iron. During the cold season, with low sedimentation rates and a sufficient supply of oxygen or nitrate to the sediments, a high redox potential is established, maintaining the sedimentary iron in its oxidized form.

A typical feature of the seasonal P retention seems to be reduced negative retention in early summer (Fig. 5). This probably relates to the clearwater phase as a consequence of late-spring development of a high zooplankton biomass and its grazing on phytoplankton[26,94,95,96]. The coupling between clearwater conditions and decreasing internal loading most likely involves several mechanisms. Reduced sedimentation of organic matter reduces oxygen consumption and enhances redox conditions, and improved light conditions increase benthic primary production and with it the uptake of P and oxidation of the sediment surface[97]. Biomanipulation experiments involving a reduction of the zooplanktivorous fish biomass and, as a result, improved transparency have often been observed to lead to decreased in-lake P. Such observations significantly emphasize the importance of clearwater conditions for decreasing internal P loading in shallow lakes (see later).

## LONGEVITY OF INTERNAL LOADING

The longevity and importance of internal loading for overall lake P-concentrations relate mainly to the flushing rate, loading history, and chemical characteristics of the sediment[12]. A high flushing rate may ensure a rapid return to low in-lake P concentrations after the external loading reduction provided that the period with high P loading was relatively short. On the other hand, a long loading history with a high loading rate is reflected in the size of the P pool accumulated in the sediment, and, if large, a rapid flushing rate may not suffice to ensure a fast return to low concentrations[13]. The chemical characteristics of the sediment including concentrations and forms of P are important for the size of the releasable P pool but also for the degradability of sedimentary organic matter which serves as an energetic basis for microorganisms. Traditionally, P in the upper ~10 cm is considered to take part in the whole lake metabolism[22], but mobility of P from depths down to 20–25 cm has also been indicated (Fig. 6). Most likely the sediment depth interacting with the lake water is lake specific and highly dependent on lake morphology, sediment characteristics, and wind exposure.

Due to different loading histories and lake characteristics the recovery period after reduced external loading varies considerably among lakes. Some lakes respond rapidly to an external loading reduction and in-lake concentrations immediately or only with a short delay follow the changes in loading[11,28,64,98,99]. Chemical resistance following nutrient loading reduction has, however, been observed in many lakes[12,13,16] and the release may be very persistent and endure for many years, a conservative estimate of at least 10 years after an external loading reduction was effected[100]. In some lakes, P retention can remain negative even longer after the nutrient loading reduction (Fig. 7).

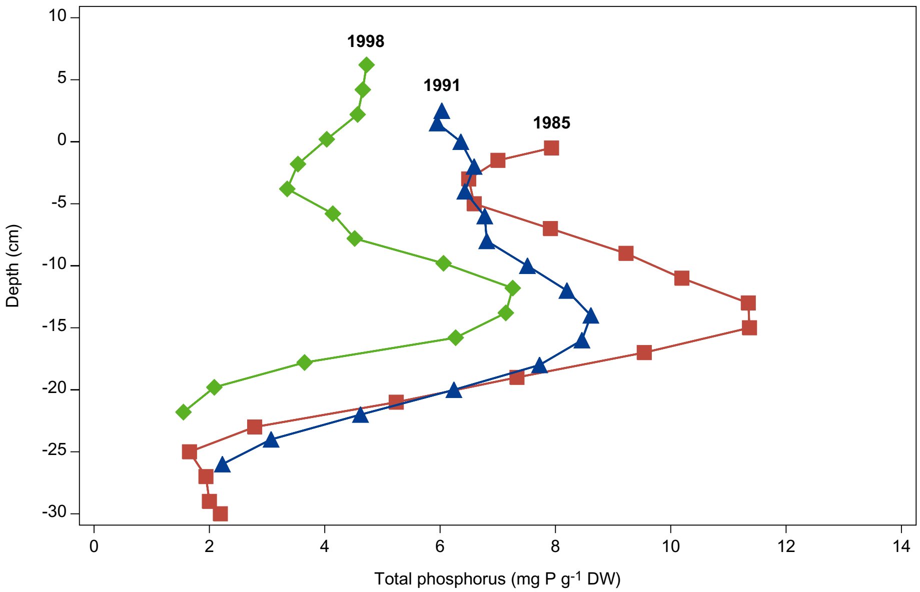

**FIGURE 6.** Sediment profiles of total phosphorus ($TP_{sed}$) in Lake Søbygaard in 1985 (square), 1991 (triangle), and 1998 (star). The sediment phosphorus profile of Lake Søbygaard changed markedly during 13 years. In the upper 25–30 cm of the sediment, $TP_{sed}$ has decreased at all depths. During the first 6 years, phosphorus was primarily released from the very high concentrations found at 15–20 cm depth, but during the latter 7 years $TP_{sed}$ has decreased at all depths. At most depths down to 25-30 cm, $TP_{sed}$ has been reduced by 3–4 mg P $g^{-1}$ DW. Calculations based on comparisons of the 1985 and 1998 profiles show that a total of 57 g P $m^{-2}$ has been released from the upper 20 cm sediment. In the same period, mass balance measurements show a total release of approximately 40 g P $m^{-2}$. At the present release rate this means that another 15–20 years will pass before the lake will be in equilibrium, implying that the transient phase after reduced external loading in total will last for more than 30 years. Sediment cores were sampled from a central location and sectioned into 2-cm slices. Sediment from three different cores was pooled into one sample before analysis (see also Søndergaard et al.[58]). Sediment profiles were adjusted to the 1985 level using a sedimentation rate of 0.6 cm $y^{-1}$. From Søndergaard, M., Jensen, J.P., and Jeppesen, E. (1999) Internal phosphorus loading in shallow Danish lakes. *Hydrobiologia* **408/409**, 145-152. With kind permission from Kluwer Academic Publishers. URL: http://www.wkap.nl

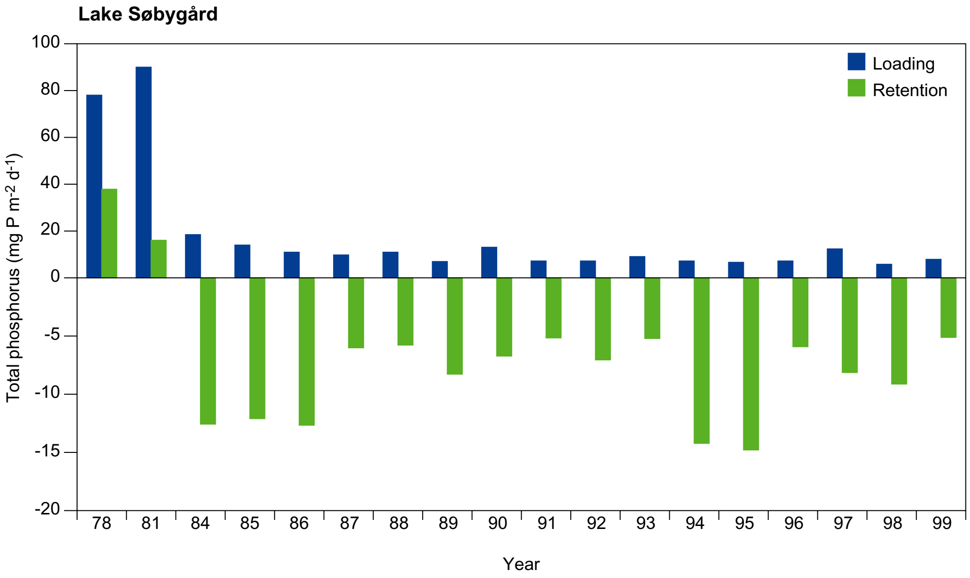

**FIGURE 7.** External loading and P retention in Lake Søbygaard from 1978 to 1999. Lake Søbygaard (area 40 ha, mean depth 1.2 m, water retention time 3–4 weeks) was heavily loaded with wastewater for several decades[46,58,126]. In the 1970s and early 1980s, annual loading was about 30 g P $m^{-2}$ $y^{-1}$. In 1982, chemical treatment was implemented and external P loading consequently decreased by 80–90%. Since 1982, mean summer P has ranged from 0.4 to 1.0 mg P $l^{-1}$ and chlorophyll *a* between 130 and 840 µg $l^{-1}$[130]. Note different scale on y-axis.

During the phase of recovery, both the duration and net P release rates from the sediment seem to decline progressively.

## IMPORTANCE OF BIOLOGICAL STRUCTURE

Changes in the biological structure of a lake significantly influence P concentrations and retention[99]. Clearwater conditions resulting from either increased top-down control on phytoplankton or increased abundance of submerged macrophytes often ensure considerably lower in-lake nutrient concentrations[101,102,103,104]. The presence of a positive relationship between clearwater conditions and increased P retention is supported by observations from biomanipulated lakes (Fig. 8). In such lakes increased retention capacity of P both prolongs the period of positive P retention in the sediment and also diminishes the summer net release rates. This observation is in agreement with earlier findings recording both considerably lower P and nitrogen concentrations after a change in biological structure[104,105,106].

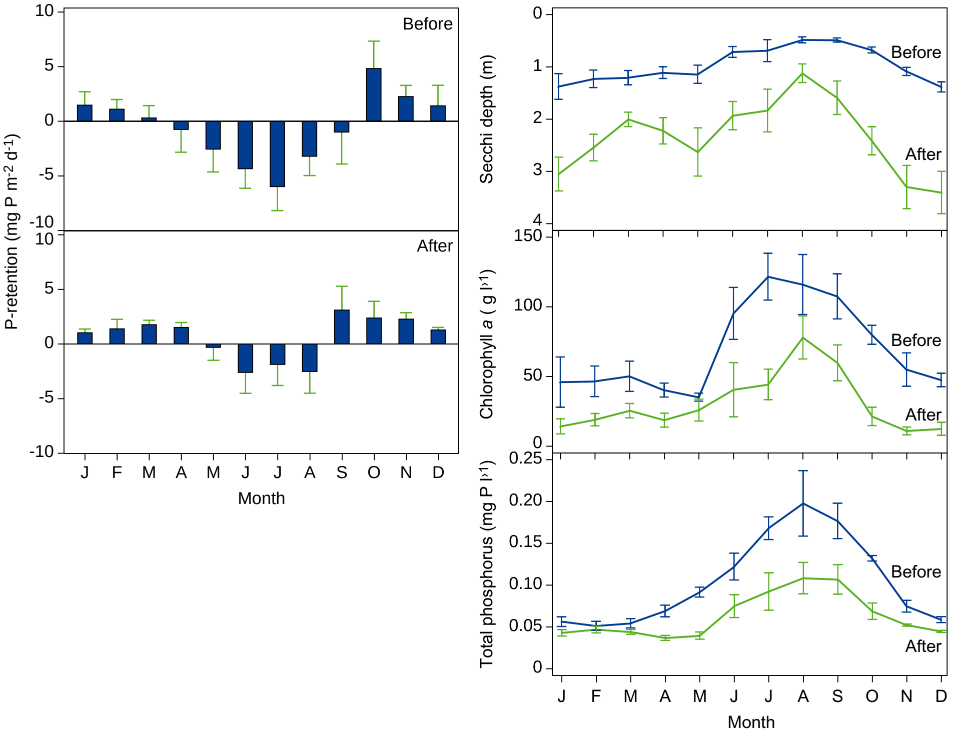

**FIGURE 8.** *Right:* Mean Secchi depth, chlorophyll *a*, and total phosphorus (TP); *left:* monthly retention of TP (±SE) in Lake Engelsholm before and after biomanipulation. Mean summer TP decreased from 0.15 to 0.07 mg P $l^{-1}$, whereas winter TP was unaffected. Both the duration and levels of seasonal TP retention changed. Prior to biomanipulation, P retention was negative for six months (April to September), but after biomanipulation the period with negative retention declined to four months (May to August). Minimum mean retention, –6 mg P $m^{-2}$ $d^{-1}$ (July), was observed before biomanipulation, being only –2 mg P $m^{-2}$ $d^{-1}$ (June and August) after the intervention. Mean TP retention in winter was unchanged and varied, except for a high retention in October before the manipulation, between 1 and 3 mg P mg P $m^{-2}$ $d^{-1}$ both before and after the manipulation. The lake was biomanipulated from April 1992 to September 1994 by removing cyprinid fish, mainly bream (*Abramis brama*), which constituted 85% of the total catch, and roach (*Rutilus rutilus*). In total, 19.2 t, or 438 kg wet weight $ha^{-1}$, were removed, corresponding to 66% of the total fish stock estimated for 1990[104,131]. Pre-conditions refer to the years 1989–1993 and post-conditions to 1994–1999. External TP loading remained unchanged during the investigation period. Marked changes in biological structure were recorded after the cyprinid reduction and a significant increase in number and size of *Daphnia* and a marked reduction in chlorophyll *a* were recorded[101]. Secchi depth increased from a summer level of 0.5–0.6 m to 1.2–2.0 m, while chlorophyll *a* decreased from 90–120 μg $l^{-1}$ to 35–70 μg $l^{-1}$. The changes were recorded throughout the season, including winter when Secchi depth increased from less than 1.5 m to more than 3 m and chlorophyll *a* decreased from about 50 μg $l^{-1}$ to about 10 μg $l^{-1}$.

Several mechanisms behind increased P retention when shallow lakes shift from the turbid to the clearwater state may be involved, including those affecting seasonal retention (see above). If submerged macrophytes become abundant they have a positive influence on lake water clarity[107,108,109]. This can affect the retention of P, both because the macrophytes assimilate P and because increased water clarity leads to enhanced benthic algal production that may reduce P release from the sediment. The reduction in the abundance of benthivorous fish (e.g., bream (*Abramis brama*)) has a significant impact on the resuspension of sediment[110] as well as on TP concentrations, and the release of P from the sediment[67,111] may be a factor of particular importance in biomanipulated lakes formerly dominated by bream or other benthivorous species[106,112]. The effects on lake water quality of fish-mediated P release from the sediment are sometimes believed to be stronger than those achieved through reduced planktivory and top-down control on phytoplankton[113,114].

## METHODS TO OVERCOME INTERNAL LOADING

In recent decades numerous lake restoration techniques have been developed and tested in an attempt to reduce internal P loading and accelerate lake recovery after decreased external loading[61,100,115,116,117,118,119]. They include both physical measures such as sediment dredging by which nutrient-rich sediment is removed and chemical methods. The chemical methods aim to influence the redox-dependent P fixation achieved either by improving the sorption capacity of the elements already present in the lake/sediment or by adding new sorption capacity. For all types of restoration measures, an important prerequisite for obtaining success and long-term effects is elimination of the underlying reasons for the undesirable water quality, i.e., a sufficient reduction of external P loading[106,119,120,121].

P control by increasing the existing sorption capacity is most often used in stratified lakes and usually obtained by the use of oxygen or less often nitrate to improve the redox-dependent P sorption[36,122,123,124]. P control by increasing the sorption capacity has been used in shallow lakes and is usually obtained by the use of iron or alum, or, more rarely, calcium to increase the formation of calcite and the precipitation of P into hydroxyapatite[100,122,125,126,127]. Alum treatment has the advantage over iron to establish a non-redox sensitive binding of P to aluminum hydroxides.

## ACKNOWLEDGMENT

This work was partly financed by the EU-project BUFFER. The technical staff at the National Environmental Research Institute, Silkeborg, are gratefully acknowledged for their assistance. Field and laboratory assistance was provided by J. Stougaard-Pedersen, B. Laustsen, L. Hansen, L. Nørgaard, K. Jensen and L. Sortkjær. Layout and manuscript assistance was provided by A.M. Poulsen, P. Nygaard, A.D. Hansen, and T. Christensen. Data were partly collected and made available by local county authorities. We wish to thank K. Havens for inviting us to write this review.
